# White Matter Integrity of Age‐Related Hearing Loss and Cognitive Impairment

**DOI:** 10.1002/alz.093247

**Published:** 2025-01-09

**Authors:** Samaneh Nemati, Meisam Arjmandi, Roger Newman‐Norlund, Sarah Newman‐Norlund, Julius Fridriksson

**Affiliations:** ^1^ University of South Carolina, Columbia, SC USA

## Abstract

**Background:**

Emerging research, underscored by the UK National Institute of Health and Care Excellence and the US National Institutes of Health, suggests that age‐related hearing loss (ARHL) is linked to the development of dementias such as Alzheimer's disease (AD). In this study, we aim to investigate the neural correlates of ARHL and cognitive impairments based on the changes in white matter (WM) microstructures in a population of non‐demented adults.

**Method:**

129 participants (94 female) aged between 20‐79 years old (Mean = 51.35 ± 20.65) were recruited as part of the Aging Brain Cohort at the University of South Carolina (ABC@UofSC). Cognitive performance was assessed using the Montreal Cognitive Assessment (MoCA). Hearing ability was evaluated by measuring pure‐tone thresholds (PTT) and words‐in‐noise (WIN) thresholds. Structural T1 weighted and diffusion tensor imaging (DTI) scans were administered, and data preprocessing was performed with our nii_preprocess automated pipeline, available for public use. Changes in the WM integrity were measured using fractional anisotropy (FA) and mean diffusivity (MD). Finally, we examined the relationship between DTI metrics (FA and MD) and hearing and cognitive performance using region‐of‐interest‐based regression analysis.

**Result:**

Our investigation identified a constellation of brain regions where lower FA and higher MD were significantly associated with both poorer auditory and cognitive processing. Specifically, poorer cognitive performance, as reflected by lower MoCA total scores, was associated with lower FA and higher MD values across major interconnecting pathways, including superior longitudinal fasciculus, fornix, corona radiata, and splenium of corpus callosum. Individuals with poorer hearing sensitivity and difficulty in words‐in‐noise recognition showed reduced FA and increased MD in fornix, superior longitudinal fasciculus, optic tract, and corona radiata (anterior and posterior sections).

**Conclusion:**

Our findings reveal that the integrity of the white matter tracts across multiple brain regions is correlated with both auditory and cognitive performance, suggesting that these tracts may underlie a shared neural substrate for hearing and cognitive processes. The identified brain regions offer promising neural targets for the early detection of ARHL and cognitive impairments, as well as potential neural substrates for applying effective intervention strategies aimed at reducing the risk of dementia.

